# SEX DIMORPHISM IN AGING

**DOI:** 10.1093/geroni/igac059.656

**Published:** 2022-12-20

**Authors:** Bérénice Benayoun

## Abstract

In this symposium, the speakers will discuss how sex can influence aging trajectories using complementary perspectives and approaches across multiple biological scales and systems. Indeed, accumulating evidence across species has revealed that aging is a highly sex-dimorphic process. On the one hand, women outlive men consistently across populations, and most supercentanarians are women. On the other hand, especially after the onset of menopause, women are at increased risk for most age-related diseases (e.g. Alzheimer’s disease, osteoporosis, etc.). However, the molecular pathways underlying such sex-differences in aging and longevity are still largely unexplored and poorly understood. Dr. Austad will discuss how compatibility between the mitochondrial and nuclear genomes during aging is influenced as a function of sex. Dr. Dubal will discuss the impact of sex chromosome complement on cognition with aging in mouse models, and how sex chromosomes may underlie sex-differences in brain aging. Dr. Ucar will discuss work from her lab revealing that the genomic signatures of immune aging in humans are specific to sex. Finally, Dr. Benayoun will discuss how aging of innate immune cells (e.g. macrophages or neutrophils) is regulated by the female vs. male milieu, with sex-specific age-related trajectories remodeling the immune compartment in mice.

